# Expanding Access to HIV Viral Load Testing: A Systematic Review of RNA Stability in EDTA Tubes and PPT beyond Current Time and Temperature Thresholds

**DOI:** 10.1371/journal.pone.0113813

**Published:** 2014-12-01

**Authors:** Kimberly Bonner, Reed A. Siemieniuk, Andrew Boozary, Teri Roberts, Emmanuel Fajardo, Jennifer Cohn

**Affiliations:** 1 Médecins Sans Frontières, Access Campaign, Geneva, Switzerland; 2 Department of Medicine, University of Toronto, Toronto, Ontario, Canada; 3 Harvard School of Public Health, Boston, MA, United States of America; 4 Médecins Sans Frontières, Southern Africa Medical Unit, Cape Town, South Africa; 5 Division of Infectious Diseases, University of Pennsylvania School of Medicine, Philadelphia, PA, United States of America; Harvard Medical School, United States of America

## Abstract

**Background:**

HIV viral load (VL) testing is the gold standard for antiretroviral treatment monitoring, but many barriers exist to VL testing in resource-limited settings, including storage and transport limitations for whole blood and plasma. Data from various studies indicate that HIV RNA is stable beyond current recommendations. We conducted a systematic review to assess stability data of HIV RNA in whole blood and plasma across times and temperatures.

**Methods and Findings:**

Using a pre-defined protocol, five databases were searched for studies where blood samples from HIV patients were stored at time and temperature points that exceeded manufacturer recommendations. RNA stability, the primary outcome, was measured by the difference in means compared to samples stored within established thresholds. RNA stability was defined as ≤0.5 log degradation. The search identified 10,716 titles, of which nine full-text articles were included for review. HIV RNA maintained stability in EDTA whole blood and plasma at all measured time points up to 168 hours when stored at 4°C, while stability was detected at 72 hours (95% confidence) in whole blood at 25°C, with data points before and beyond 72 hours suggesting stability but not reaching statistical significance. For EDTA plasma stored at 30°C, stability was maintained up to 48 hours (95% confidence), with OLS linear regression estimates up to 127 hours, suggesting stability. Overall, quality of studies was moderate. Limitations included small sample sizes, few studies meeting inclusion criteria, and no studies examining RNA stability in low viremia (<3,000 copies/mL) environments.

**Conclusions:**

Whole blood and plasma samples in EDTA may remain stable under conditions exceeding current manufacturer recommendations for HIV VL testing. However, given the limited number of studies addressing this question, especially at low levels of viremia, additional evaluations on HIV RNA stability in EDTA tubes and PPT in field conditions are needed.

## Introduction

The 2013 WHO HIV treatment guidelines strongly recommend routine viral load (VL) testing six and twelve months after treatment initiation and then at least every year thereafter (strong recommendation, low-quality evidence) [1]. VL testing enables clinicians to monitor the efficacy of antiretroviral therapy and to identify patients struggling with treatment adherence before they develop resistance. Identifying elevated VLs early and providing enhanced adherence support can lead to over 70% of patients successfully re-suppressing HIV viral replication to undetectable levels [2].

Despite the benefits of VL testing, HIV RNA sample transport constraints limit testing access in resource-limited settings. It is estimated that only 20% of people on antiretroviral treatment (ART) in Africa access routine VL testing [3].

For VL testing, samples may be shipped to the laboratory as whole blood, plasma, or dried blood or plasma spots (DBS/DPS), depending on the resources available. Optimizing sample collection options depends on the trade-offs between test accuracy and logistical capacity.

DBS/DPS remain stable over extended time and temperature periods, often making this the only practical transport option in remote areas. However, the smaller sample volume reduces sensitivity and the cell-associated nucleic acid can reduce specificity [4], [5]. For this reason, testing plasma samples is preferred, where feasible.

Plasma transport with ethylenediaminetetraacetic acid (EDTA) tubes or plasma preparation tubes (PPT) is the gold standard for VL testing. However, generating plasma samples requires trained phlebotomists and electricity in health posts to power the centrifuges. Current recommendations for plasma storage and transport require that plasma is transported within 24 hours at 25°C in EDTA tubes, or within 5 days at 4°C for EDTA tubes or PPT, after centrifugation (Appendix S1) [6]. If there is no access to centrifugation, whole blood in EDTA tubes or PPT cannot be stored more than 6 hours at 25°C. The logistical constraints, coupled with overall expense, make this option challenging to implement in resource-limited settings. Although phlebotomists are still required for venous blood sampling, whole blood transport relieves the need of electricity for centrifugation in health posts – a requirement 30% of health posts in Sub-Saharan Africa lack – as well as potential transcription errors, and safety and contamination risks, associated with plasma processing [7].

The limited sample transport time and temperature thresholds effectively limit the radius of whole blood transport to 6 hours at 25°C or to 24 hours at 4°C. The implications of such cautious labelling would not significantly affect care in developed countries, since maintaining the cold chain is less difficult, and there is widespread access to centrifuges and efficient specimen transport services. However, in resource-poor environments, such restrictive guidance on whole blood and plasma transport greatly limits access to VL testing to only those in close proximity to national or regional laboratories. This systematic review seeks to measure HIV RNA stability in EDTA tubes and PPT at extended time and temperature thresholds, compared to the established thresholds, in all published studies.

## Methods

Using a pre-defined protocol, five databases (Pubmed, EMBASE, Ovid, LILACS, and Web of Science) were searched according to pre-specified search terms (Appendix S2). No restrictions on language, publication status, or year of publication were used. Titles were accessed from database inception to August 13^th^, 2013 or October 28^th^, 2013, depending on database.

After an initial title search, selected abstracts were reviewed in duplicate using piloted forms. Any disagreements were resolved with a third reviewer. The same procedure was followed for inclusion of full text articles and data extraction. Articles were included if they measured HIV RNA stability beyond the established time and temperature thresholds for whole blood or plasma with either EDTA tubes or PPT. Extracted information included characteristics of the study, population, intervention, outcome and risk of bias. Recommended time and temperature thresholds include: <6 hours at room temperature (defined as 25°C) for whole blood in EDTA tubes or PPT and <24 hours in whole blood in EDTA at 4°C. Plasma can be stored for 24 hours at room temperature (defined as 25°C) in EDTA tubes or 5 days at 4°C for EDTA tubes or PPT.

Risk of bias was estimated for each study according to a modified Jadad scale adapted for basic science [8]. Risk of bias was then summarized according to the GRADE framework [9], [10].

The primary outcome measured was mean log declines in RNA stability beyond recommended time and temperature thresholds, with significant declines in RNA stability reported as a secondary outcome. Data was analyzed by mean VL differences at different temperature thresholds. Mean and standard errors were calculated from median and range when not otherwise provided [11]. When not provided in the study, standard deviation of the difference in means was imputed using the Cochrane Handbook methodology and the coefficients of correlation provided among the included studies [12]. Corresponding authors were contacted for any additional information needed.

A viral degradation cutoff of > 0.5 log copies/mL was used to define clinical significance, consistent with the literature [13], [14]. Figures were developed to chart RNA stability over time by transport type at specific temperature thresholds, with lines of best fit generated using Stata 13.

## Results

**Figure 1:**
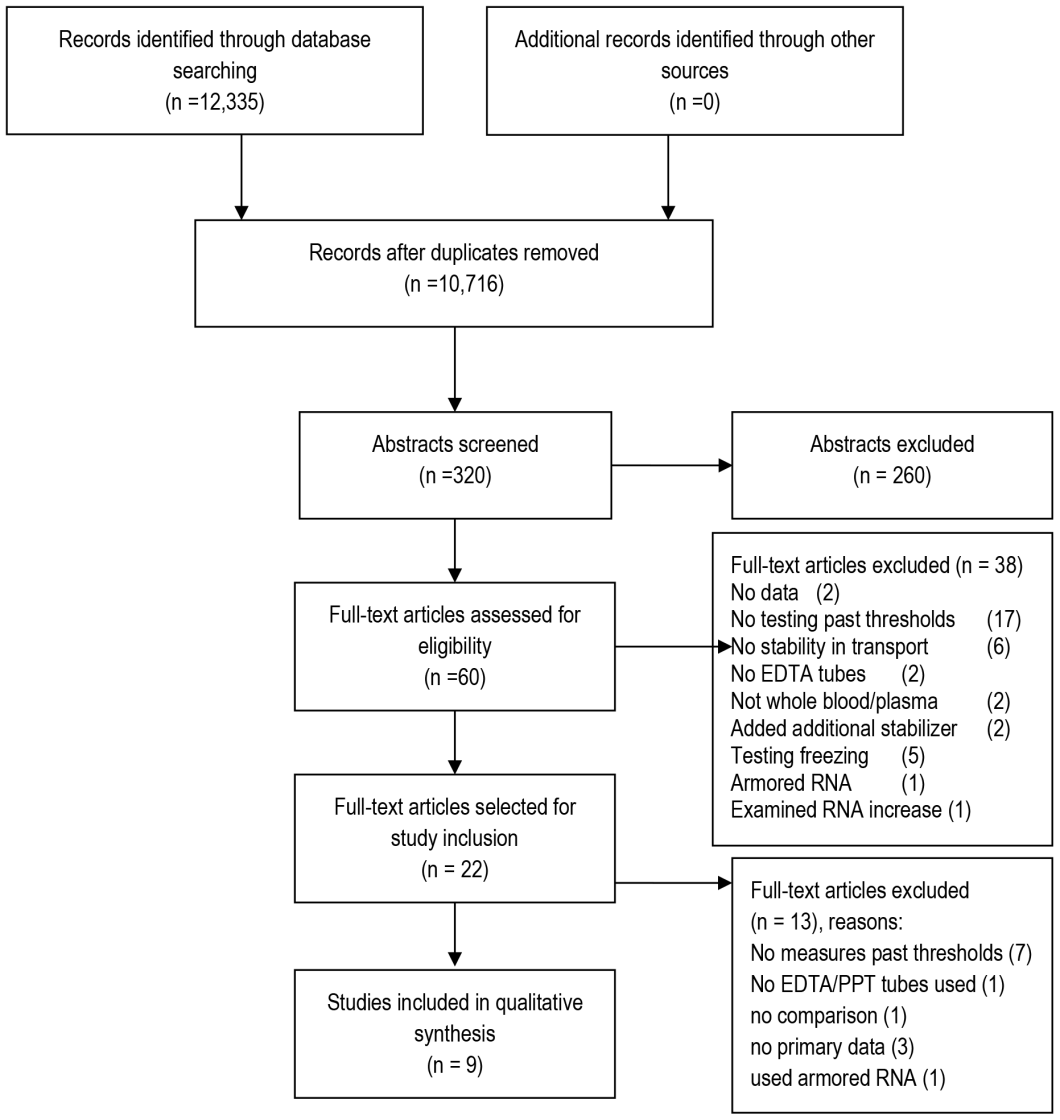
Search Strategy.

The database search yielded 10,716 unique titles (Figure 1); 60 articles were reviewed in full text and nine studies were taken through for review. Characteristics of selected studies are outlined in Table 1.

**Table 1:** Characteristics of included studies.

Author	Year	Design	Population	N	Tube	Assay	Intervention	Control	Outcome	
Amellal [22]	2007	Matched controlled trial	Mixed treatment population, France	10	EDTA	Roche Amplicor HIV-1 Monitor v1.5	Plasma>1 d 25°C,	Plasma 80°C	7d 4°C rho = .98, p = .003	
							Plasma>5 d 4°C		7d 22°C rho = .99, p<.001	
									7d 37°C rho = .87, p = .012	
Amellal [21]	2008	Matched controlled trial	HIV+ on ART, France	25	EDTA	Roche Cobas Taqman HIV-1	Plasma>5 d 4°C,	Plasma 7d 20°C	7d 4°C rho = .98, p = .003	
							22°C, 30°C, 37°C		7d 22°C rho = .99, p = <.001	
									7d 30°C rho = .99, p = <.001	
									7d 37°C -.92 log copies (p = .05)	
Bruistein [15]	1997	Matched controlled trial	ART naïve, Netherlands	14	EDTA	NucliSens HIV-1 QT	Whole blood>6h 25°C	Matched blood samples at t = 0.	Whole blood (median, range) log copies)	Plasma 30°C (median, range)
							Whole blood>1 d 4°C		0h 4°C 5.99 (4.46–6.59)	0d 5.19 (4.70–5.78)
							Plasma>1 d 25°C		6h 4°C 5.93 (4.53–6.39)	1d 5.19 (4.74–5.68)
							Plasma>5 d 4°C		1d 4°C 5.87 (4.39–6.26	2d 5.07 (4.56–5.68)
									3d 4°C 5.87 (4.26–6.23)	7d 4.45 (4.04–5.18)
									0h 25°C 6.01 (4.39–6.53)	14d 3.59 (3.30–4.33)
									6h 25°C 5.90 (4.55–6.59)	
									1d 25°C 5.93 (4.35–6.56)	
									3d 25°C 5.93 (4.48–6.54)	
Dickover [16]	1998	Matched controlled trial	Mixed treatment population, USA	20	EDTA	Roche Amplicor HIV-1 Monitor	Whole blood>6h 25°C	<1 h 25°C EDTA	1d 25°C 80% (+/.08) RNA baseline	
									2d 25°C 72% (+/.08) RNA baseline	
Gessoni [17]	2004	Matched controlled trial	ART naïve, Italy	25	EDTA	Roche Cobas Amplicor HIV-1 Monitor	Whole blood>1 d 4°C	Whole blood Room temp for 3h	<6h 4°C 4.677 log copies	
									7d 4°C 4.33 log copies	
Holguin [18]	1997	Matched controlled trial	HIV+, pre-HAART era	29	EDTA	Roche Cobas Amplicor HIV-1 Monitor	Whole blood>24h Room temp	Whole blood Room temp for 4h	24h room temperature 3.671 log copies; *P* = 0.082 vs. control*	
Holodiny [23]	1995	Matched controlled trial	ART naïve, USA	6	PPT	Siemens Versant HIV-1 RNA (bDNA)	Whole blood>6h 25°C		30h 137% (+/48 SE) RNA baseline, not significant	
Kirstein [19]	1999	Matched controlled trial	Mixed treatment population, USA	65	EDTA	Siemens Versant HIV-1 RNA; Roche Cobas Amplicor HIV-1 Monitor	Whole blood>6h 25°C	Whole blood Room temp for 2h	6h 23°C -.02 (+/.14 SD) RNA baseline	
									18h 23°C 0.9 (+/.17 SD) RNA baseline	
Vandamme [20]	1999	Matched controlled trial	HIV+, Belgium, Luxembourg	12	EDTA	NucliSens HIV-1 QT	Whole blood 48 h, 168h 25°C	Whole blood t = 0	48h 25°C 0.125 log decline168h 25°C 0.296 log decline	
									168h 25°C 0.296 log decline	

ART, antiretroviral therapy; PPT, plasma preparation tube; EDTA,; rho, spearman’s rank correlation coefficient.

* Note: there was no trend towards more variation among lower viral loads (data not specified).

Studies measured RNA degradation in EDTA whole blood (n = 6)[15]–[20], EDTA plasma (n = 3) [15], [21], [22], and PPT whole blood (n = 1) [23] (Table 1). All included studies were controlled studies with matched blood samples drawn from the same individuals. Populations included ART naïve patients (n = 4) [15], [17], [18], [23] and mixed or unspecified treatment populations (n = 5) [16], [19]–[22]. All studies examined RNA stability in the laboratory, rather than field conditions. Six of the nine studies were published prior to 2000, and all were conducted in the United States and Europe. Each study reported storage conditions for both the experimental and control arm.

With regards to risk of bias, according to a modified Jadad scale (Appendix S3), all studies met the requirements for randomization, with a well-designed and completed protocol. All blood sources were derived from patients with HIV. The same diagnostic platforms and processes were used for experimental and control arms.

### EDTA whole blood

**Figure 2:**
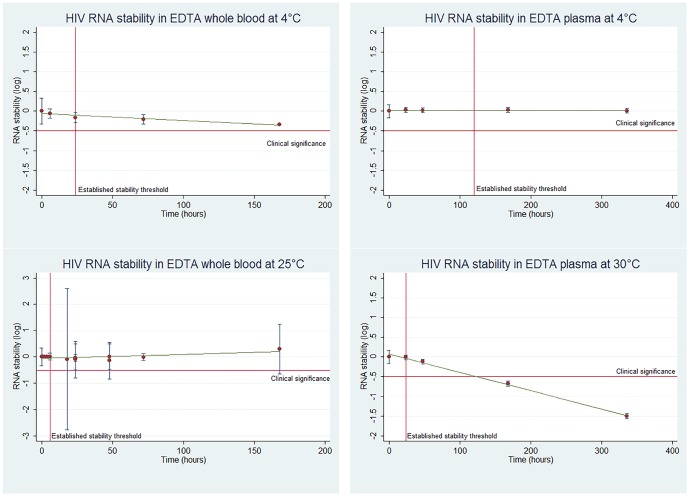
RNA degradation in EDTA tubes over time and temperature.

For EDTA whole blood, six studies assessed RNA stability at 4°C and room temperature (25°C), with time points at zero, six, 18, 24, 48, 72, and 168 hours (Table 1). HIV RNA remained stable for all measured time points until 168 hours at 4°C with 95% confidence [15]. For the EDTA whole blood samples stored at room temperature (25°C) beyond the established stability threshold, four time points were within the acceptable threshold for RNA stability, and another four had a point estimate that suggested stability, but the confidence intervals overlapped the 0.5 log degradation threshold. The latest time point to maintain statistically significant stability according to the pre-specified criteria was at 72 hours [15]. An additional study at room temperature found that 72.4% of the samples measured had less than 0.2 log degradation, with 96.5% at less than 0.5 log degradation after 24 hours [18]. The matrix of RNA stability in EDTA whole blood and plasma over time is indicated in Figure 2.

### EDTA plasma

Three studies assessed the stability of RNA in EDTA plasma, at 4°C, 22°C, 30°C and 37°C, with time points at 24, 48, 168, and 336 hours (Table 1). There was limited decline in RNA copies/mL at 4°C after 168 hours (0.04 log copies/mL) and after 336 hours (0.01 log copies/mL), with 95% confidence [15], [21], [22].

At 30°C, HIV RNA maintained stability in EDTA plasma until 48 hours (0.12 log reduction). At 168 hours there was a significant decline of 0.74 log copies/mL, with continued declines at 336 hours (1.6 log copies/mL decline) [15], [21]. Assuming a linear degradation of RNA in these samples, and using Ordinary Least Squares (OLS) linear regression, the stability threshold was surpassed after 127 hours, though this analysis is limited by the lack of data between 48 and 168 hours and by the fact that there is little data to assume a linear pattern of degradation under these conditions. After 168 hours at 37°C, the difference in means was 0.92 log copies/mL [22]. At 22°C, there was limited (0.03 log copies/mL) and insignificant decline after 168 hours. Due to insufficient time points at 22°C and 37°C, no figures are presented with results from these EDTA samples.

### PPT whole blood

Only one study assessed the stability of PPT whole blood. It found a no significant degradation 30 hours post collection at 25°C [23]. Instead, there was a 137% increase in the baseline RNA copies/mL.

The studies were evaluated under a GRADE framework and found to be of moderate quality, owing to the small sample sizes and failure to exclude publication bias (Appendix S4).

## Discussion

Our analysis indicates that HIV RNA is stable in EDTA tubes beyond the current manufacturer recommendations for time and temperature and highlights the need for more definitive studies at higher temperatures that may be encountered in countries with high HIV burdens. For EDTA whole blood transport, RNA remains stable until 72 hours, with data points at times beyond 72 hours suggesting stability, but with 95% confidence intervals that spanned the stability threshold. For EDTA plasma at 30°C, measures of RNA were within the stability threshold at 48 hours. While a meta-regression suggested stability beyond 48 hours (estimated stability until 127 hours), no data points were available between 48 and 168 hours and one cannot assume linear RNA degradation under these conditions. More data is needed to confirm the true stability of EDTA plasma at 30°C beyond 48 hours. These studies indicate that transport for both EDTA whole blood and plasma can potentially be greatly expanded, thus increasing the feasibility of transporting blood tubes to laboratories in resource limited settings.

Acceptable RNA degradation over the course of sample transport needs to be assessed through the lens of clinical relevance. A VL in whole blood or plasma of 1,000 copies/mL is recommended by the 2013 consolidated WHO HIV treatment guidelines as the threshold by which to define virological failure [1]. Thus, in certain cases where VLs fall close to the 1,000 copies/mL threshold, a decline of>0.5 log in RNA stability may lead to incorrect clinical management decisions. According to a multi-country VL assessment of 21,909 adults, only 4.4% had VL results between 1,000–5,000 copies/mL in routine VL testing, with an even smaller percentage falling within 0.5 log of the failure threshold [24]. Further clarity on acceptable thresholds for degradation is needed, particularly at lower (<3,000 copies/mL) VL values. In our analysis, we considered declines of greater than 0.5 log copies/mL to be clinically relevant, consistent with the literature.

The precedent for expanded sample transport thresholds have already been set by the British HIV Association. In their 2011 guidelines for routine monitoring, EDTA whole blood storage for 2–3 days at room temperature is recommended, with evidence cited from at least one well-designed quasi-experimental study [25]. However, this recommendation would not account for temperatures over 30°C, which are rarely seen in the UK, but are frequent in many low- and middle-income countries.

Adapting sample transport regulations to match the demonstrated HIV RNA stabilities would enable VL sample collection to expand and reach more people living with HIV. A one-week sample transport window would allow for bi-weekly or weekly sample collection, provided samples were kept at 25°C, as might be possible with passive cooling devices, such as a cooler box.

Countries implementing VL testing in resource–limited settings will need guidance on acceptable time and temperature thresholds for sample transport, particularly in hot climates. Given that the studies included in this systematic review indicate that stability at extended time and temperature thresholds is greater than current recommendations indicate, we recommend that suppliers of EDTA tubes and PPT, as well as viral load tests, urgently undertake the necessary studies to allow for more flexible sample transport recommendations.

### Limitations

Some of these studies are not sufficiently powered to assess significance at 95% confidence at particular timepoints. Much of this variability may be attributed to variation across viral loads in patient samples, rather than over time. As only aggregate data was available, a matched pair comparison of differences in HIV RNA stability over time was not possible, thus confidence intervals around the point estimates are conservative estimates.

The majority of included studies were published prior to 2000 and the Roche Amplicor HIV-1 Monitor was the most utilized VL assay. While the age of these studies would not impact the RNA stability, newer VL technologies may better detect RNA and could narrow the RNA degradation gap. Our study did not incorporate the variability in precision between VL assays, although these have been documented [26]. For example, the new Roche COBAS Ampliprep/COBAS TaqMan HIV-1 v2.0 assay has been reported to overestimate HIV RNA levels in plasma compared to other technologies [26]. Most studies were adequately powered to account for these low-magnitude variations.

These studies were laboratory-based, rather than examining the field impacts of sample transport beyond time and temperature thresholds, with only one study examining PPT transport. Given the logistical constraint of centrifugation, the added value of PPT may be limited if whole blood remains stable in EDTA tubes for extended periods. Additionally the inconsistent assessments of time and temperature thresholds limited comparability across studies.

The impact of sample transport conditions on low and undetectable VLs need to be further examined. The majority of people in these studies had VLs over 3.5 log copies/mL with some not receiving ART. Populations accessing VL monitoring today are generally receiving ART, and VLs in these populations are lower than those not on ART [1]. A 2010 systematic review of virological suppression in adults in sub-Saharan Africa found that 78% had virologically suppressed after 6 months, 76% after 12 months and 67% after 24 months of treatment [27]. While adherence is generally lower among adolescents and young adults, a recent systematic review found that adherence was 84% (95% CI 79–89%) in this population in Sub-Saharan Africa [28]. Assessments of HIV RNA stability drawn from these largely undetectable and low viremia populations will be needed for insights on RNA stability at low levels and the potential impact of even minor RNA degradation on clinical decision-making for these populations.

This systematic review examined RNA stability over time and temperature, but it did not examine increases in HIV RNA past time and temperature thresholds. Extended storage time may erroneously detect intracellular viral particles after cell lysis, which is of unknown clinical significance [29]. Given this theoretical risk, and the lack of studies particularly for patients with undetectable VLs, we caution generalization of our findings to this population. Further studies are warranted to establish the safety of extended storage conditions when monitoring patients on ART.

## Conclusions

All published studies show that HIV RNA has an extended time and temperature stability compared to current product labelling. Further studies are needed to determine the precise threshold of expansion and to correlate possible time and temperature-induced increases on HIV RNA at low VLs, especially with the newest VL technologies. This information is urgently needed in order to revise sample transport guidelines and facilitate the expansion of HIV VL monitoring in low- and middle-income countries.

## Supporting Information

Appendix S1 **Standard thresholds by manufacturer.**
(DOCX)

Appendix S2 **Review Protocol.**
(DOC)

Appendix S3 **Risk of bias adapted Jadad Scale.**
(DOCX)

Appendix S4 **GRADE Analysis.**
(DOCX)

Appendix S5 **Stata meta-regression command and output.**
(DOCX)

Checklist S1 **PRISMA 2009 Checklist.**
(DOC)
