# Supplementary material for: Expanding Access to HIV Viral Load Testing: A Systematic Review of RNA Stability in EDTA Tubes and PPT beyond Current Time and Temperature Thresholds
Source: PLoS One. 2014 Dec 1;9(12):e113813. doi: 10.1371/journal.pone.0113813 (PMC4249975; doi:10.1371/journal.pone.0113813)
Supplement: Appendix S1 — Standard thresholds by manufacturer. (DOCX) [file pone.0113813.s001.docx]

Appendix S1. Standard thresholds by manufacturer

| **Company** | **Assay** | **EDTA Whole Blood** | **EDTA Plasma** |
| --- | --- | --- | --- |
| Roche | COBAS Ampliprep/COBAS Taqman | *6h at 2-25°C* | *5 days at 2-8°C*  *24h at RT* |
| Abbott | RealTi*me* HIV-1 | *6h at 15-30°C*  *24h at 2-8°C* | *5 days at 2-8°C*  *24h at 15-30°C*  60 days at -20°C |
| bioMérieux | NucliSENS EasyQ HIV-1 v2.0 | *24h at 2-8°C* | 7 days at 2-8°C  1 month at -20°C  1 year at -70°C |

| **Company** | **Assay** | **PPT Whole Blood** | **PPT Plasma** |
| --- | --- | --- | --- |
| Roche | COBAS Ampliprep/COBAS Taqman | *6h at 2-25°C* | *5 days at 2-8°C*  24h at RT |
| Abbott | RealTi*me* HIV-1 | *6h at 2-25°C* |  |

All italicized thresholds were included in the search
