# Supplementary material for: Expanding Access to HIV Viral Load Testing: A Systematic Review of RNA Stability in EDTA Tubes and PPT beyond Current Time and Temperature Thresholds
Source: PLoS One. 2014 Dec 1;9(12):e113813. doi: 10.1371/journal.pone.0113813 (PMC4249975; doi:10.1371/journal.pone.0113813)
Supplement: Appendix S2 — Review Protocol. (DOC) [file pone.0113813.s002.doc]

**Appendix S2. Review Protocol**

**HIV RNA Stability in Whole Blood versus Plasma for measurement of HIV viral load among patients with HIV-1 infection**

**Question: How far beyond the time and temperature thresholds for EDTA and PPT plasma and whole blood does HIV RNA remain stable?**

**SEARCH STRATEGY**

**Databases**

- Cochrane Infectious Diseases Group Specialized Register
- Cochrane Central Register of Controlled Trials
- MEDLINE
- EMBASE
- CABS Abstracts
- LILACS
- Web of Science

**Restrictions**

No date or language restriction will be applied.

**INCLUSION CRITERIA**

**Types of studies**[[1]](#footnote-2)

- All studies that report on paired viral load measures before and after 1). six hours at storage 15-25°C or 2). after 24 hours at storage 2-8°C or RNA degradation beyond these thresholds.

**Types of participants**

Inclusions:

- All humans living with HIV (including pediatric populations), diagnosed based on a prior serologic test
- Any geographic area

**Types of outcomes**

*Primary*

- Correlation between the pre and post threshold paired measures using Bland-Altman plot.

*Secondary*

- RNA degradation measures post-threshold as compared to standard degradation at 1). 6 hours room temperature or 2). 24 hours 2-8°C.

**APPENDIX**

| **Search 1 pubmed: accessed all publication dates through August 13^th^, 2013**  1.HIV Infections OR HIV OR hiv OR hiv-1* OR hiv-2* OR hiv1 OR hiv2 OR hiv infect* OR human immunodeficiency virus OR human immunedeficiency virus OR human immuno-deficiency virus OR human immune-deficiency virus OR ((human immun*) AND (deficiency virus)) OR acquired immunodeficiency syndrome OR acquired immunedeficiency syndrome OR acquired immuno-deficiency syndrome OR acquired immune-deficiency syndrome OR ((acquired immun*) AND (deficiency syndrome)) OR "sexually transmitted diseases, Viral”  2.RNA OR RNA, viral OR RNA, virus, Viral load OR ribonucleic acid  3.Handle OR handling OR handled OR collect OR collection OR collected OR store OR storage OR storing OR stable OR stability OR stabilities OR process OR processing OR processed OR transit OR shipping OR ship OR shipped OR temperature  Search #1 AND #2 AND #3 |
| --- |
| **Search 2 EMBASE: accessed all publication dates through Aug 14^th^, 2013**  1. 'hiv'/de OR hiv AND infections OR 'hiv'/de OR hiv OR 'hiv 1'/de OR 'hiv 1' OR 'hiv 2'/de OR 'hiv 2' OR 'hiv1'/de OR hiv1 OR 'hiv2'/de OR hiv2 OR ('hiv'/de OR hiv AND infect) OR ('human'/de OR human AND ('immunodeficiency'/de OR immunodeficiency) AND ('virus'/de OR virus)) OR ('human'/de OR human AND immunedeficiency AND ('virus'/de OR virus)) OR ('human'/de OR human AND 'immuno deficiency' AND ('virus'/de OR virus)) OR ('human'/de OR human AND ('immune deficiency'/de OR 'immune deficiency') AND ('virus'/de OR virus)) OR (acquired AND ('immunodeficiency'/de OR immunodeficiency) AND ('syndrome'/de OR syndrome)) OR (acquired AND immunedeficiency AND ('syndrome'/de OR syndrome)) OR (acquired AND 'immuno deficiency' AND ('syndrome'/de OR syndrome)) OR (acquired AND ('immune deficiency'/de OR 'immune deficiency') AND ('syndrome'/de OR syndrome)) OR (sexually AND transmitted AND diseases, AND viral)  2. 'rna'/exp/mj OR 'rna' OR rna, AND viral OR rna, AND virus, AND viral AND load OR ribonucleic AND ('acid'/exp/mj OR 'acid')  3. handle OR handling OR handled OR collect OR collection OR collected OR store OR 'storage'/exp/mj OR storage OR storing OR stable OR stability OR stabilities OR process OR 'processing'/exp/mj OR processing OR processed OR transit OR 'shipping'/exp/mj OR shipping OR 'ship'/exp/mj OR ship OR shipped OR 'temperature'/exp/mj OR temperature  Search #1 AND #2 AND #3 |
| **Search 3 OVID (Biosis & CABS abstracts): accessed all publications through October 28^th^, 2013**   1. (HIV Infections OR HIV OR hiv OR hiv-1 OR hiv-2 OR hiv1 OR hiv2 OR (hiv infect) OR (human immunodeficiency virus) OR (human immunedeficiency virus) OR (human immuno-deficiency virus) OR (human immune-deficiency virus) OR (human immune deficiency virus) OR (acquired immunodeficiency syndrome) OR (acquired immunedeficiency syndrome) OR (acquired immuno-deficiency syndrome) OR (acquired immune-deficiency syndrome) OR (acquired immune deficiency syndrome) OR (sexually transmitted diseases, Viral)) 2. (RNA OR (RNA, viral) OR (RNA, virus) OR (Viral load) OR (ribonucleic acid)) 3. (Handle OR handling OR handled OR collect OR collection OR collected OR store OR storage OR storing OR stable OR stability OR stabilities OR process OR processing OR processed OR transit OR shipping OR ship OR shipped OR temperature)   Search #1 AND #2 AND #3 |
| **Search 4 LILACS: accessed all publications through October 23, 2013**  1. tw:(HIV)  2. tw:(RNA OR viral load)  3. tw:(Handle OR handling OR handled OR collect OR collection OR collected OR store OR storage OR storing OR stable OR stability OR stabilities OR process OR processing OR processed OR transit OR shipping OR ship OR shipped OR temperature)  4. Search #1 AND #2 AND #3 |
| **Search 5 Web of Science: accessed all publications through October 23, 2013**  1. Topic=(HIV)  2. Topic=(RNA)  3. Topic=(handle OR handling OR handled OR collect OR collection OR collected OR store OR storage OR storing OR stable OR stability OR stabilities OR process OR processing OR processed OR transit OR shipping OR ship OR shipped OR temperature)  4. Search #1 AND #2 AND #3 |

1. <http://www.ispor.org/TaskForces/documents/ProspectiveObservationalStudiesGRPDraftCurrent.pdf> [↑](#footnote-ref-2)
