# Supplementary material for: Expanding Access to HIV Viral Load Testing: A Systematic Review of RNA Stability in EDTA Tubes and PPT beyond Current Time and Temperature Thresholds
Source: PLoS One. 2014 Dec 1;9(12):e113813. doi: 10.1371/journal.pone.0113813 (PMC4249975; doi:10.1371/journal.pone.0113813)
Supplement: Appendix S3 — Risk of bias adapted Jadad Scale. (DOCX) [file pone.0113813.s003.docx]

Appendix S3. Risk of bias adapted Jadad Scale

| Study | Year | Randomization (add or deduct 1 additional point if method of randomization is appropriate/inappropriate)) | Protocol well-defined (replicable) | Blood source (spiked blood moderate quality) | Completion (Samples in control & experimental arm are traced to the conclusion of the study) | Standardization (both arms used the same diagnostic platforms and processes) |
| --- | --- | --- | --- | --- | --- | --- |
| Amellal | 2007 | 1 | Yes | High | 1 | 1 |
| Amellal | 2008 | 1 | Yes | High | 1 | 1 |
| Bruistein | 1997 | 1 | Yes | High | 1 | 1 |
| Dickover | 1998 | 1 | Yes | High | 1 | 1 |
| Gessoni | 2004 | 1 | Yes | High | 1 | 1 |
| Holodiny | 1995 | 1 | Yes | High | 1 | 1 |
| Holguin | 1997 | 1 | Yes | High | 1 | 1 |
| Kirstein | 1999 | 1 | Yes | High | 1 | 1 |
| Vandamme | 1999 | 1 | Yes | High | 1 | 1 |
