# Supplementary material for: Expanding Access to HIV Viral Load Testing: A Systematic Review of RNA Stability in EDTA Tubes and PPT beyond Current Time and Temperature Thresholds
Source: PLoS One. 2014 Dec 1;9(12):e113813. doi: 10.1371/journal.pone.0113813 (PMC4249975; doi:10.1371/journal.pone.0113813)
Supplement: Appendix S4 — GRADE Analysis. (DOCX) [file pone.0113813.s004.docx]

Appendix S4. GRADE Analysis

| Comparison | Outcome | Quantity and Type of Evidence | Findings | Starting GRADE | Decrease GRADE | | | | | Increase GRADE | | | GRADE of Evidence for Outcome | Overall GRADE |
| --- | --- | --- | --- | --- | --- | --- | --- | --- | --- | --- | --- | --- | --- | --- |
|  |  |  |  |  | Study quality | Consistency | Directness | Precision | Publication Bias* | Large Magnitude | Dose-response | Confounders |  |  |
| What is the impact of storing EDTA in whole blood or plasma at higher temperatures or longer time periods | | | | | | | | | | | | | |  |
| HIV RNA in EDTA/PPT before established threshold vs. HIV RNA in EDTA/PPT after established threshold | log decline in HIV RNA | Matched Controlled Study | Amellal 2007, Amellal 2008, Bruistein 1997, Dickover 1998, Gessoni 2004, Holguin 1997, Kirstein 1999, Vandamme 1999 | High | 0 | 0 | 0 | -1 (Few samples in each study/threshold)* | Cannot exclude | 0 | 1 (expected temperature and time response) | 0 | Moderate | Moderate |

|  | significant decline in RNA | Matched Controlled Study | Holodiny 1995 | High | 0 | 0 | 0 | -1 (Few samples in each study/threshold)* | - 1 Cannot exclude | 0 | 0 | 0 | Moderate |  |
| --- | --- | --- | --- | --- | --- | --- | --- | --- | --- | --- | --- | --- | --- | --- |

* These studies are not industry-funded and no language restriction was used. However, the sample sizes are small. A funnel plot was not utilized due to the variety of temperature and time thresholds
