# Supplementary material for: Expanding Access to HIV Viral Load Testing: A Systematic Review of RNA Stability in EDTA Tubes and PPT beyond Current Time and Temperature Thresholds
Source: PLoS One. 2014 Dec 1;9(12):e113813. doi: 10.1371/journal.pone.0113813 (PMC4249975; doi:10.1371/journal.pone.0113813)
Supplement: Appendix S5 — Stata meta-regression command and output. (DOCX) [file pone.0113813.s005.docx]

Appendix S5. Stata meta-regression command and output

Meta-regression of the difference in means in EDTA plasma at 30°C

Stata command

metareg DifferenceinMeans Time if WBPlasma ==0 & Temp ==30, wsse(Standard_error_of_the_difference_in_means)

Log for Figure 2. RNA degradation in EDTA tubes over time and temperature

clear

use "C:\Users\kbonner\Documents\Viral load\RNA stability FINAL.dta"

log using "C:\Users\kbonner\Documents\Viral load\RNA stability final.smcl", replace

*With alternative cochrane equation for SD

*plasma

twoway rcap Cochrane_Up Cochrane_Low Time if WBPlasma ==0 & Temp ==4 || scatter DifferenceinMeans Time if WBPlasma ==0 & Temp ==4|| lfit DifferenceinMeans Time if WBPlasma ==0 & Temp ==4

twoway rcap Cochrane_Up Cochrane_Low Time if WBPlasma ==0 & Temp ==30 || scatter DifferenceinMeans Time if WBPlasma ==0 & Temp ==30|| lfit DifferenceinMeans Time if WBPlasma ==0 & Temp ==30

twoway rcap Cochrane_Up Cochrane_Low Time if WBPlasma ==0 & Temp ==25 || scatter DifferenceinMeans Time if WBPlasma ==0 & Temp ==25|| lfit DifferenceinMeans Time if WBPlasma ==0 & Temp ==25

*Whole blood

twoway rcap Cochrane_Up Cochrane_Low Time if WBPlasma ==1 & Temp ==4 || scatter DifferenceinMeans Time if WBPlasma ==1 & Temp ==4|| lfit DifferenceinMeans Time if WBPlasma ==1 & Temp ==4

twoway rcap Cochrane_Up Cochrane_Low Time if WBPlasma ==1 & Temp ==30 || scatter DifferenceinMeans Time if WBPlasma ==1 & Temp ==30|| lfit DifferenceinMeans Time if WBPlasma ==1 & Temp ==30

twoway rcap Cochrane_Up Cochrane_Low Time if WBPlasma ==1 & Temp ==25 || scatter DifferenceinMeans Time if WBPlasma ==1 & Temp ==25|| lfit DifferenceinMeans Time if WBPlasma ==1 & Temp ==25

*OLS linear Reg

reg DifferenceinMeans Time if WBPlasma ==0 & Temp ==30

*Metareg reference formula"

*metareg depvar [indepvars] [if] [in] wsse(varname) [, eform graphrandomsize noconstant mm reml eb knapphartung z tau2test level(#) permute(# [, univariable detail joint(varlist1 [| varlist2 ...])]) log maximize_options]"

metareg DifferenceinMeans Time if WBPlasma ==0 & Temp ==30, wsse(Standard_error_of_the_difference_in_means)

log close
